# Characterising the severity of treatment resistance in unipolar and bipolar depression

**DOI:** 10.1192/bjo.2021.1004

**Published:** 2021-10-13

**Authors:** Rachael W. Taylor, Rebecca Strawbridge, Allan H. Young, Roland Zahn, Anthony J. Cleare

**Affiliations:** The Centre for Affective Disorders, Department of Psychological Medicine, Institute of Psychiatry Psychology and Neuroscience, King's College London, UK; The Centre for Affective Disorders, Department of Psychological Medicine, Institute of Psychiatry Psychology and Neuroscience, King's College London, UK; and South London and Maudsley NHD Foundation Trust, UK

**Keywords:** Depressive disorders, in-patient treatment, out-patient treatment, rating scales, individual psychotherapy

## Abstract

**Background:**

Treatment-resistant depression (TRD) is classically defined according to the number of suboptimal antidepressant responses experienced, but multidimensional assessments of TRD are emerging and may confer some advantages. Patient characteristics have been identified as risk factors for TRD but may also be associated with TRD severity. The identification of individuals at risk of severe TRD would support appropriate prioritisation of intensive and specialist treatments.

**Aims:**

To determine whether TRD risk factors are associated with TRD severity when assessed multidimensionally using the Maudsley Staging Method (MSM), and univariately as the number of antidepressant non-responses, across three cohorts of individuals with depression.

**Method:**

Three cohorts of individuals without significant TRD, with established TRD and with severe TRD, were assessed (*n* = 528). Preselected characteristics were included in linear regressions to determine their association with each outcome.

**Results:**

Participants with more severe TRD according to the MSM had a lower age at onset, fewer depressive episodes and more physical comorbidities. These associations were not consistent across cohorts. The number of episodes was associated with the number of antidepressant treatment failures, but the direction of association varied across the cohorts studied.

**Conclusions:**

Several risk factors for TRD were associated with the severity of resistance according to the MSM. Fewer were associated with the raw number of inadequate antidepressant responses. Multidimensional definitions may be more useful for identifying patients at risk of severe TRD. The inconsistency of associations across cohorts has potential implications for the characterisation of TRD.

## Treatment resistance – definitions and impact

Treatment-resistant depression (TRD) is a significant clinical problem, despite the availability of a broad range of efficacious treatments.^1^ The prevalence of early-stage resistance in primary care has been reported to be 55% of patients prescribed one adequate antidepressant treatment for depression,^2^ and TRD has a 40% higher annual direct cost compared with non-TRD, correlating with the severity of resistance.^3^ The extensive burden of TRD also includes poorer long-term outcomes.^4^ Patients with TRD therefore represent a vulnerable group in need of additional support.

Despite the prevalence of antidepressant resistance, there is no consensus definition of TRD. Approximately six characterisations have been used in the research literature, and this has contributed to variation in controlled treatment trials and treatment guidelines for TRD, which recommend the use of next-step strategies for TRD at varying stages of illness.^5,6^ The European Medicines Agency guidelines for the treatment of depression operationalise TRD as a lack of clinically meaningful improvement with adequate treatment trials of at least two different antidepressant agents of the same or different classes, but recognise that validated clinical criteria and thresholds to define TRD are not currently available.^7^ This limits the comparability of existing research, consistency in TRD treatment and the coherence of future research aims.

Patients with TRD are commonly operationally defined as a distinct clinical group (i.e. those meeting specific criteria according to the number of failed treatments), but there is evidence that TRD should be considered as a continuum, beginning at (risk of) one treatment failure and increasing in severity.^8^ Currently, the National Institute for Health and Care Excellence recommend augmentation at stage three of their stepped-care pathway following non-/insufficient response to ≥2 antidepressant treatment trials in the current depressive episode,^9^ but evidence that remission rates fall with each subsequent antidepressant treatment^10^ means that the ability to identify not only risk of TRD, but risk of TRD severity would facilitate a more nuanced approach to the recommendation and implementation of such next-step treatment pathways.

The use of individual patient characteristics to identify individuals at risk of severe TRD would allow those patients to be prioritised for the more intensive and specialist treatment options at an earlier stage in their illness trajectory. Given that recommended next-step treatments are known to be efficacious among patients with TRD,^1^ their appropriate early application to those at risk of severe TRD has the potential to minimise the occurrence and severity of resistance. In order to achieve this aim, characteristics associated with the severity of treatment resistance must first be identified.

## Measures of treatment resistance

A recent review by Salloum & Papakostas identified five staging models for TRD.^8^ Despite differences between the five staging models, including variation in the number and type of treatments undertaken, and the definition of inadequate response, four of the measures were unidimensional assessments of TRD, whereby the number of treatment ‘failures’ is used to determine the presence of TRD and its severity. The Maudsley Staging Method (MSM) was the only multidimensional measure identified. In addition, characterising TRD according to the number of treatment failures, which includes pharmacological augmentation and electroconvulsive therapy as well as antidepressants, the MSM also accounts for current depression severity and duration of the current depressive episode in the total score.^11^ The inclusion of the severity and duration dimensions provides more nuanced information about the specific nature of the depression and both have been identified as risk factors for TRD.^12–16^

The MSM has been shown to be predictive of subsequent treatment outcomes^13^ but the inclusion of severity and episode duration has given rise to some criticism, including suggestions that the MSM is not a pure assessment of TRD as treatment-naive patients can score above zero.^8^ However, such scores may indicate a vulnerability to resistance, and therefore have both clinical and research relevance, particularly when further assessing the association between patient characteristics and treatment resistance.

## Characteristics associated with treatment resistance

Considerable research to date has assessed predictors of (or risk factors for) TRD additional to severity and episode duration.^13^ In a recent large-scale study of patients in the Danish National Patient Registry, illness severity factors (severity, recurrence and admissions) were strong risk factors for TRD when defined as two suboptimal treatment responses, as well as older age, unemployment, cohabiting with a partner, anxiety, insomnia, migraine and psychotropic medication use.^17^ Many of these factors have also been identified in previous, smaller assessments of TRD risk factors, as well as psychotic symptoms,^18^ earlier age at onset, melancholic features, bipolarity, experience of childhood trauma and personality traits.^18–21^

Understanding whether these factors also predict the severity of treatment resistance would indicate whether they can be used to identify patients at risk of persistent and severe non-response. Assessing the association between TRD risk factors and TRD severity according to both univariate and multivariate assessments of TRD demonstrate if one is likely to have greater clinical utility for this purpose than the other, and clarify if TRD is appropriately characterised as a continuous construct.

## Study aims and objectives

This study aimed to assess whether individual-level characteristics can explain the degree of treatment resistance in patients with depression according to a common unidimensional definition (the raw number of antidepressant treatment failures within an episode), and according to the only validated multidimensional TRD measure (the MSM). This will indicate whether established risk factors for TRD can be used to identify patients at risk of severe treatment resistance, and whether this is best achieved using a univariate or multivariate characterisation of TRD.

To do this, three cohorts of patients were evaluated:

- patients without established treatment resistance in primary care (those receiving treatment via Improving Access to Psychological Therapies (IAPT) services);
- patients with TRD who had failed to respond to ≥2 antidepressant treatments for unipolar depression and therefore were eligible for secondary care (for example out-patient mental health services); and
- patients with a greater severity of established resistance admitted for specialist treatment in a tertiary care service (the affective disorders unit in-patient service at the Bethlem Royal Hospital).

Patient characteristics were selected according to existing evidence that they are risk factors for TRD and therefore it was hypothesised that they would be associated with the degree of treatment resistance when assessed both multidimensionally and as the number of antidepressant failures. We hypothesised that these characteristics would show broadly similar associations across treatment cohorts (where comparison is possible), indicating a consistency across the TRD spectrum, and would show similar associations with each outcome measure.

## Method

We examined data from cross-sectional baseline (pre-treatment) assessments across three cohorts of adult patients (≥18 years) with unipolar or bipolar depression:

- the Predicting Outcome following Psychological Therapy (PROMPT) study;
- the Lithium versus Quetiapine in Depression (LQD) study; and
- the Affective Disorders Unit (ADU) studies.^22–24^

### Ethical approval

All studies had ethical approval and written informed consent was obtained from all participants.

The authors assert that all procedures contributing to this work comply with the ethical standards of the relevant national and institutional committees on human experimentation and with the Helsinki Declaration of 1975, as revised in 2008. All procedures involving human patients were approved by the following ethics committees. LQD: The Cambridge South Research Ethics Committee (REC), UK (registration number: 16/EE/0318), the Medicines and Healthcare products Regulatory Authority (MHRA: EudraCT: 2016-001637-27) and the Health Research Authority. PROMPT: The Bromley NHS REC (reference 13/LO/1347). ADU: Institute of Psychiatry ethics committee (reference 285/03 and 322/03).

### PROMPT participants

Patients accepted into the UK's IAPT service (Southwark borough, London) and who provided their written informed consent were eligible for participation in the PROMPT study and the following inclusion criteria were applied in line with the primary analyses. The full study protocol has been previously published:^22^

- ≤1 session within the service before PROMPT baseline assessment;
- attended ≥2 sessions of low or high intensity therapy;
- Patient Health Questionnaire (PHQ) score ≥10 at first therapy session;^25^
- ≤20% missing data for relevant independent variables;
- available outcome data for the relevant analysis (MSM score or number of antidepressant treatment failures).

### LQD participants

Participants in the LQD study, with relevant outcome data available (MSM total score/number of antidepressant failures) were included in the present analyses. LQD inclusion criteria are detailed in the published protocol,^23^ and included the following key characteristics:

- inadequate response to ≥2 antidepressant therapies in the current episode;
- met criteria for current depressive episode according to DSM-5;^26^
- Hamilton Rating Scale for Depression (HRSD-17) score ≥14 at screening;^27^
- no diagnosis of bipolar disorder or current psychosis according to the DSM-5;
- no adequate treatment trial (current episode) or ongoing use of lithium or quetiapine augmentation. Other atypical antipsychotic use was also not permitted at baseline.

### ADU participants

Patients admitted to the ADU, a specialist tertiary care in-patient service at the Bethlam Royal Hospital between 2001 and 2012, and who provided written informed consent, were eligible for participation. In line with previous work using data from the ADU studies,^28^ the following inclusion criteria were applied:

- primary diagnosis of an affective disorder (ICD-10);^29^
- HRSD-21 score ≥16 at admission;
- failure to adequately respond to at least one pharmacological treatment trial;
- ≤20% missing data for relevant independent variables;
- available outcome data for the relevant analysis.

### Outcome variables

The primary outcome was MSM total score.^13^ The MSM is a multidimensional measure of treatment resistance in which three components contribute to a single total score: number of treatment non-responses, depression severity and episode chronicity.^11^ The secondary outcome was the number of antidepressant treatment failures (current episode).

### Predictor variables

Clinical and sociodemographic characteristics were selected for inclusion based on existing evidence of an association with treatment outcome. As a result of the secondary nature of these analyses data were collected cross-sectionally. Therefore, we did not include characteristics that were likely to be highly dynamic across the adult lifespan, such as depression severity. Predictor variables were matched across cohorts as far as possible:

- age at depression onset (PROMPT, LQD, ADU);
- number of previous depressive episodes (PROMPT, LQD, ADU);
- childhood trauma (PROMPT– Childhood Trauma Questionnaire (CTQ),^30^ ADU –presence/absence of trauma^31^);
- Standardised Assessment of Personality – Abbreviated version (SAPAS)^32^ (PROMPT, LQD);
- psychiatric comorbidities (PROMPT – Mini International Diagnostic Interview (MINI),^33^ LQD – medical records/self-report, ADU – clinical admission data);
- bipolarity (PROMPT – MINI, ADU – clinical assessment);
- marital status (PROMPT, LQD, ADU);
- physical illness (PROMPT – Cumulative Illness Rating Scale (CIRS),^34^ LQD – presence/absence in medical records/self-report, ADU – presence/absence in clinical admission assessment);
- duration of illness (PROMPT, LQD, ADU);
- lifetime psychosis (PROMPT – MINI criteria indicating a current or lifetime psychotic disorder or mood disorder with psychotic features, LQD – MINI criteria indicating a lifetime psychotic disorder or mood disorder with psychotic features, ADU – clinical assessment indicating the presence or history of psychotic symptoms within a mood episode);
- melancholic depression (PROMPT – MINI);
- years of education (PROMPT, LQD, ADU);
- atypical depression (LQD – Inventory of Depressive Symptomatology (IDS)^35^);
- family history (ADU);
- gender (PROMPT, ADU, LQD).

### Statistical analyses

#### Data pre-processing and imputation

For all cohorts, data were cleaned using SPSS version 25 and R version 3.6. Missing data for the predictor variables were imputed using the Multivariate Imputation by Chained Equations (MICE) approach, via the MICE package in R. This method creates multiple imputations for missing continuous and categorical data by running multiple regressions in which the missing values in a variable are modelled, conditional on the other variables in the data-set, producing multiple complete data-sets according to the number of cycles specified.^36^

#### Regression analyses

To assess the association of patient characteristics with severity of treatment resistance (MSM score or number of treatments trialled), linear multiple regressions were used using the R package GLM. Each study data-set (PROMPT, LQD and ADU) was assessed separately for the primary and secondary outcomes because of the heterogeneous nature of the patient groups and differences in the data collected for each study.

For each analysis, MICE imputation produced several complete data-sets on which the relevant regression was run. The results from the five data-sets were then pooled to give model parameters and regression coefficients, as is recommended practice following MICE imputation.^37,38^ For all models in this study data were checked for high leverage points, normality of the residuals, heteroskedasticity and multicollinearity (Supplementary Tables 1–6 available at https://doi.org/10.1192/bjo.2021.1004).

## Results

### Participant characteristics

Patient characteristics for each of the data-sets included in this study are shown in Table 1 and Supplementary Table 7. Participants in the ADU cohort were the most highly treatment resistant according to both outcome measures, followed by the LQD participants. PROMPT participants had the lowest MSM scores and fewest treatment failures. The ADU and PROMPT cohorts predominantly consisted of patients with unipolar depression (72% and 73%, respectively), whereas all participants in the LQD study cohort had a diagnosis of unipolar depression (Table 1).

**Table 1:** Participant characteristics

	PROMPT cohort			LQD cohort^a^			ADU cohort		
	*n*	Median (IQR)	%	*n*	Median (IQR)	%	*n*	Median (IQR)	%
Age, years	139	36.0 (21.5)		198	42.8 (22.1)		170	50.0 (18.8)	−
Gender	139			198	−			−	
Female		−	69.1			54.5	170		74.1
Male			30.9			45.5			25.9
Ethnicity	106	–		197	−			−	
White			70.8			89.9	167		97.0
Black			3.8			2.0			1.2
Asian			11.3			4.6			1.8
Other			14.1			3.5			0.0
Marital group^b^	132	−		197	−			−	
Single			51.5			47.2	170		20.0
Separated			7.6			8.6			63.5
Steady			40.9			44.2			16.5
Education	132	−		198					
Years or categories^c^			1: 11.4		15.0 (3.0)	−	166	13.0 (5.0)	−
			2: 13.6						
			3: 20.5						
			4: 54.5						
Age at onset, years	139	16.0 (12.0)	−	197	17.0 (15.0)	−	170	27.0 (22.0)	−
Duration of illness, years	139	16.0 (15.5)	−	198	18.9 (17.7)	−	170	15.0 (20.0)	−
Previous episodes, *n*	139	−		197	−			−	
0			28.1			2.7	167		22.8
1			15.8			34.6			18.0
2			7.2			21.3			13.2
≥3			48.9			41.4			46.1
Diagnosis	139	−		198	−			−	
Unipolar			72.7			100	170		71.8
Bipolar			27.3			0			28.2
Melancholic subtype	132	−		−	−		−	−	
No			50.8			−			−
Yes			49.2						
Lifetime psychosis	132	−		−	−		170	−	
No			89.1			−			61.8
Yes			10.9						38.2
Atypical subtype				197	−		−	−	
No	−	−	−			92.4			−
Yes						7.6			
Psychiatric comorbidities, *n*	138	1.0 (1.0)	−	197	2.0 (2.0)	−	163	0.0 (1.0)	
Family history^d^	−	−		−	−		164	−	
No			−			−			39.0
Possible									14.6
Definite									46.4
Physical illness	138			195	−		160	−	
Yes		15.0 (3.8)^e^	−			83.1			72.5
No						16.9			27.5
Childhood trauma	129			−	−		165	−	
Yes		38.0 (23.0)^f^	−			−			58.8
No									41.2
Personality (SAPAS) score	132	3.0 (3.0)	−	194	4.0 (2.0)	−	−	−	−
MSM score	139	5.0 (2.0)	−	198	8.0 (2.0)	−	170	11.0 (3.0)	−

PROMPT, Predicting Outcome following Psychological Therapy; LQD, Lithium versus Quetiapine in Depression; ADU, Affective Disorders Unit; IQR, interquartile range; SAPAS, Standardised Assessment of Personality – Abbreviated version; MSM - Maudsley Staging Method.

a. Participants recruited from sites in London (37%), North East (26%), Oxford (26%), Brighton (7%), Bristol (5%).

b. Marital group categorisation: steady, long-term relationship, cohabiting, married; separated,  divorced, marriage separated, widowed; single,  otherwise.

c. Education categories, 1, no qualifications; 2, secondary; 3, college; 4, ≥degree.

d. First-degree relative with affective disorder.

e. Cumulative Illness Rating Scale score.

f. Childhood Trauma Questionnaire score.

### PROMPT cohort – MSM outcome

In total, 139 participants were eligible for inclusion in this model. There were 120 participants with complete data before imputation and all variables had <20% missingness (highest, childhood trauma, 7.2%). The models had a pooled *R*^2^ of 0.3 (*P* < 0.001, likelihood ratio chi-square (LR χ^2^) = 3.0). The presence of a physical illness and meeting criteria for melancholic depression significantly contributed to the variance in MSM score (positive associations, Table 2).

**Table 2:** Pooled results for multivariate linear models, Maudsley Staging Method (MSM) outcome

	PROMPT			LQD			ADU		
	β (s.e.)	*t*	*P*	β (s.e.)	*t*	*P*	β (s.e.)	*t*	*P*
Intercept	3.65 (1.03)	3.52	<0.001	9.22 (0.90)	10.23	<0.001	9.44 (1.18)	7.98	<0.001
Gender	0.07 (0.23)	0.30	0.764	0.07 (0.19)	0.36	0.721	0.01 (0.36)	0.02	0.980
Marital group^a^ (separated)	−0.33 (0.48)	−0.69	0.494	0.64 (0.36)	1.77	0.077	0.24 (0.44)	1.56	0.578
Marital group^a^ (steady)	−0.09 (0.22)	−0.41	0.683	−0.13 (0.20)	−0.65	0.517	0.79 (0.55)	1.43	0.156
Education	−0.22 (0.12)	−1.88	0.062	0.03 (0.04)	0.74	0.461	−0.06 (0.04)	−1.38	0.169
Age at depression onset	<0.01 (0.01)	0.12	0.904	−0.02 (0.01)	−2.47	0.014*	0.03 (0.01)	1.69	0.094
Duration of depressive illness	<0.01 (0.01)	0.31	0.760	0.01 (0.01)	0.88	0.377	0.02 (0.02)	1.36	0.177
Previous depressive episodes	−0.13 (0.09)	−0.45	0.150	−0.53 (0.11)	−5.09	<0.001**	0.17 (0.17)	0.95	0.342
Bipolar depression	−0.09 (0.28)	−0.33	0.742	−	−	−	−0.98 (0.41)	−2.37	0.019*
Melancholic depression	0.61 (0.23)	2.68	0.009**	−	−	−	−	−	−
Lifetime psychosis	0.21 (0.38)	0.54	0.589	−	−	−	0.64 (0.32)	0.99	0.049*
Psychiatric comorbidities	0.10 (0.14)	0.72	0.472	0.06 (0.06)	1.05	0.294	0.19 (0.23)	0.84	0.404
Physical illness	0.09 (0.04)	2.17	0.032*	0.26 (0.25)	0.05	0.293	0.21 (0.36)	0.58	0.562
Childhood trauma (CTQ)	<0.01 (<0.01)	1.00	0.320	−	−	−	−0.25 (0.32)	−0.76	0.450
Personality (SAPAS)	0.08 (0.08)	1.01	0.315	−0.06 (0.07)	−0.79	0.428	−	−	−
Atypical depression (IDS)	−	−	−	−0.23 (0.37)	−0.37	0.535	−	−	−
Family history	−	−	−	−	−	−	0.11 (0.18)	0.64	0.523

PROMPT, Predicting Outcome following Psychological Therapy; LQD, Lithium versus Quetiapine in Depression; ADU, Affective Disorders Unit; s.e., standard error; CTQ – Childhood Trauma Questionnaire, SAPAS – Standardised Assessment of Personality - Abbreviated Scale; IDS, Inventory of Depressive Symptomatology.

a. Marital group categorisation: steady, long-term relationship, cohabiting, married; separated, divorced, marriage separated, widowed; single, otherwise.

*P < 0.05, **P < 0.01.

**Fig. 1:**
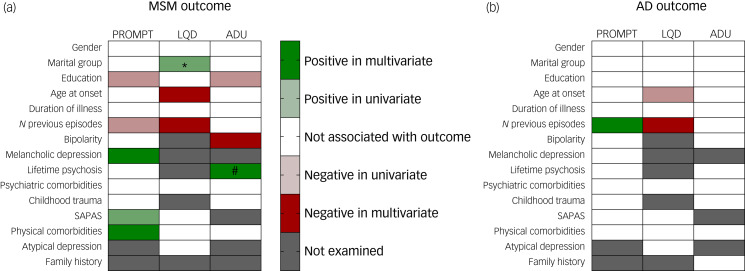
Significant associations between patient characteristics and each outcome variable. (a) Maudsley Staging Method (MSM) outcome; (b) antidepressant outcome. *Categorical predictor with three levels: in LQD, being separated/divorced/widowed associated with poor outcome on the MSM. ^#^Lifetime psychosis was not a significant predictor in univariate MSM analysis; all other variables (both outcomes) associated in multivariate regressions were also significant in univariate models. ADU, Affective Disorders Unit; LQD, Lithium versus Quetiapine in Depression; PROMPT, Predicting Outcome following Psychological Therapy; SAPAS, Standardised Assessment of Personality – Abbreviated version.

Univariate models are shown in Supplementary Table 8, and significant associations for multivariate and univariate models are shown in Fig. 1. The bivariate association between melancholic depression and MSM score is shown in Supplementary Fig. 1.

### PROMPT cohort – antidepressant trials outcome

In total, 149 participants were eligible for inclusion with the number of antidepressant treatment failures in the current depressive episode as the outcome. There were 130 participants with complete data before imputation and all variables had <20% missingness (highest, childhood trauma, 6.7%). The models had a pooled *R*^2^ of 0.1 (*P* = 0.079, LR χ^2^ = 1.6). Number of previous depressive episodes significantly (positively) contributed to the variance in antidepressant treatment failures (Table 3). Univariate models are shown in Supplementary Table 9. The bivariate association between number of previous episodes and antidepressant outcome is shown in Supplementary Fig. 2.

**Table 3:** Pooled results for multivariate linear models, antidepressants outcome

	PROMPT			LQD			ADU		
	β (s.e.)	*t*	*P*	β (s.e.)	*t*	*P*	β (s.e.)	*t*	*P*
Intercept	−0.21 (0.41)	−0.41	0.613	4.49 (0.93)	4.82	<0.01	5.37 (1.20)	2.69	0.008
Gender	0.15 (0.09)	1.57	0.118	0.20 (0.20)	0.99	0.321	0.34 (0.61)	0.56	0.577
Marital group^a^ (separated)	0.18 (0.17)	1.06	0.293	0.59 (0.37)	1.60	0.109	0.63 (0.71)	0.90	0.370
Marital group^a^ (steady)	−0.15 (0.09)	−1.62	0.107	0.04 (0.20)	0.17	0.865	0.76 (0.87)	0.87	0.387
Education	−0.01 (0.05)	−0.18	0.859	0.04 (0.04)	1.10	0.273	−0.10 (0.07)	−1.31	0.193
Age at depression onset	<0.01 (0.01)	0.10	0.924	−0.01 (0.01)	−1.49	0.136	0.01 (0.02)	0.38	0.705
Duration of depressive illness	<0.01 (<0.01)	1.09	0.277	<−0.01 (0.01)	−0.28	0.780	0.01 (0.03)	0.37	0.714
Previous depressive episodes	0.09 (0.03)	2.65	0.009**	−0.41 (0.11)	−3.76	<0.001**	0.41 (0.28)	1.50	0.135
Bipolar depression	0.02 (0.11)	0.20	0.842	−	−	−	−1.15 (0.68)	−1.69	0.093
Melancholic depression	0.14 (0.09)	1.51	0.134	−	−	−	−	−	−
Lifetime psychosis	−0.24 (0.15)	−1.62	0.107	−	−	−	−0.88 (0.53)	−1.68	0.095
Psychiatric comorbidities	0.03 (0.05)	0.52	0.603	−0.04 (0.06)	−0.56	0.575	0.003 (0.37)	0.01	0.994
Physical illness	<0.01 (0.02)	1.18	0.242	0.17 (0.25)	0.69	0.491	0.61 (0.61)	1.01	0.315
Childhood trauma (CTS)	<−0.01 (0.03)	−0.10	0.919	−	−	−	−0.88 (0.53)	−1.66	0.099
Personality (SAPAS)	−0.02 (0.03)	−0.54	0.587	−0.14 (0.07)	−1.87	0.061	−	−	−
Atypical Depression (IDS)	−	−	−	0.24 (0.39)	0.62	0.538	−	−	−
Family history	−	−	−	−	−	−	0.18 (0.29)	0.64	0.525

PROMPT, Predicting Outcome following Psychological Therapy; LQD, Lithium versus Quetiapine in Depression; ADU, Affective Disorders Unit; s.e., standard error; CTQ – Childhood Trauma Questionnaire, SAPAS – Standardised Assessment of Personality - Abbreviated Scale; IDS, Inventory of Depressive Symptomatology.

a. Marital group categorisation: steady, long-term relationship, cohabiting, married; separated, divorced, marriage separated, widowed; single, otherwise.

** *P* < 0.01.

### LQD cohort – MSM outcome

In total 198 participants from the LQD study cohort had MSM outcome data and were eligible for inclusion. There were 179 participants with complete data before imputation, and all variables had <20% missingness (highest, number of previous depressive episodes, 5.1%). Multilevel modelling was used with study site entered as a random factor. The five imputed models had a pooled *R*^2^ of 0.2 (*P* < 0.001, LR χ^2^ = 3.7). Age at onset and number of previous episodes significantly (negatively) contributed to the variance in MSM score (Table 2). Univariate models are shown in Supplementary Table 10. The bivariate association between number of previous episodes and MSM score is shown in Supplementary Fig. 3.

### LQD cohort – antidepressant trials outcome

There were 199 participants from the LQD cohort who had outcome data for this analysis and were eligible for inclusion. There were 181 participants with complete data before imputation and all variables had <20% missing data (highest, number of previous episodes, 5.0%). The models had a pooled *R*^2^ < 0.1 and were significant (LR χ^2^ = 2.2, *P* = 0.013). Pooled coefficients are shown in Table 3. Number of previous episodes was negatively associated with number of antidepressant treatment failures. Univariate models are shown in Supplementary Table 11. The bivariate association between number of previous episodes and number of antidepressant treatment failures is shown in Supplementary Fig. 4.

### ADU cohort – MSM outcome

There were 170 participants who were eligible for inclusion. There were 144 participants with complete data and all variables had <20% missingness (highest, physical illness, 5.9%). Imputation produced five complete data-sets. Pooled coefficients for the multivariate linear models are shown in Table 2. The models had a pooled *R*^2^ of 0.2 (LR χ^2^ = 2.2, *P* = 0.009). A diagnosis of bipolar depression was negatively associated with MSM score, whereas a history of psychosis was positively associated. Univariate models are shown in Supplementary Table 12. Figure 1 shows all significant associations with MSM score across cohorts.

### ADU cohort – antidepressant trials outcome

There were 180 participants who were eligible for inclusion, with number of antidepressant treatment failures as the outcome. There were 147 participants with complete data, and all variables had <20% missingness (highest,  physical illness, 7.2%). Pooled coefficients for the five multiple regressions are shown in Table 3. The models had a pooled *R*^2^ of 0.1 (LR χ^2^ = 1.5, *P* = 0.117). None of the included variables significantly contributed to the variance in number of antidepressant treatment failures. Univariate models are shown in Supplementary Table 13. Figure 1 shows all significant associations with the number of antidepressant treatment trials across cohorts.

## Discussion

This study aimed to assess whether risk factors for TRD are associated with the severity of treatment resistance in patients with depression when measured using the multidimensional MSM, and when measured unidimensionally as the raw number of antidepressant treatment failures, in three cohorts of patients with depression.

### Characteristics associated with MSM score

Melancholic depression and physical illness were both positively associated with MSM score in the PROMPT cohort of patients referred for psychological therapy, whereas higher age at onset and number of previous episodes correlated with lower levels of resistance in the LQD study cohort of TRD out-patients. Furthermore, in the ADU in-patient cohort, a diagnosis of bipolar depression was associated with lower levels of treatment resistance, but the presence of lifetime psychotic symptoms showed a positive association with MSM score. These findings clearly demonstrate that several characteristics are associated with severity of resistance when assessed using the multidimensional MSM, and it may be possible to assess risk of resistance severity using these factors. Some of the different findings between cohorts may be accounted for by differences in cohort characteristics (for example the LQD study only included patients with unipolar depression and without psychosis and therefore these characteristics were not assessed), and differences in data collection (physical illness was measured continuously using the CIRS in the PROMPT cohort, whereas the ADU and LQD cohorts used a binary physical comorbidity indicator). However, these differences may imply that the relationship between patient characteristics and TRD is not consistent across the whole continuum of TRD severity, and perhaps reflect a more complex structure to the TRD population.

Age at onset and number of previous depressive episodes were measured consistently across cohorts yet were only (negatively) associated with MSM score in the LQD multiple regression. Interestingly, the number of episodes was also negatively associated with MSM score in the PROMPT cohort when assessed univariately. This suggests that both are relevant to TRD severity in those with established, but moderate resistance, but not at the more severe end of the TRD spectrum, perhaps suggesting that patients whose condition is severely resistant represent a distinct subgroup. Age at depression onset has previously shown contrasting results in relation to resistance.^18,39^ The presence of an association with severity of resistance in TRD out-patients (LQD) but not treatment-resistant tertiary care in-patients (ADU) or primary care patients referred for psychological therapy (PROMPT) further indicates the need for additional research to understand this relationship.

Melancholic depression (according to the MINI) was associated with a higher MSM score in the PROMPT cohort, in line with previous work reporting a relationship with treatment resistance,^20^ suggesting it may indicate a vulnerability to resistance in patients without a history of non-response. Atypical depression (according to the IDS) showed no association with scores in the LQD cohort, but this may at least in part be accounted for by differences in variance, as only 7.04% of the LQD cohort were identified as having an atypical subtype, whereas 50% of the PROMPT cohort had melancholic depression. It is possible that the low proportion of patients with the atypical subtype in the LQD cohort prevented any association from being identified. However, as ~73% of PROMPT participants meeting MINI criteria for major depressive disorder also met criteria for melancholic subtype, which is higher than the expected prevalence of melancholic features,^40^ it is possible that the assessment of melancholic depression applied was oversensitive, and as not all of the PROMPT cohort met MINI criteria for major depressive disorder, the association between melancholic depression and MSM score was reflective of depression severity.

Previous reports of individuals with TRD being more likely to have a physical illness than those without TRD were supported in the PROMPT cohort, but not the LQD or ADU findings,^5^ meaning that although physical illness may indicate a vulnerability to TRD, it is not associated in patients with established TRD. However, it is possible that these results are confounded by measurement differences as previously mentioned. The CIRS used in the PROMPT cohort accounts for the severity of the physical illness in its scoring, and therefore it is possible that this weighting of physical comorbidities according to their severity is relevant to the association with TRD. This was not captured in the LQD and ADU study cohorts, for which a binary measure indicating the presence or history of a physical comorbidity was used. This is in line with previous findings reporting no association between physical illness and TRD.^41^ It is also worth noting that the CIRS contains a psychiatric illness rating, which was excluded from scores in the PROMPT cohort and therefore not driving the association reported here.

The negative association between bipolar depression and MSM score in the ADU cohort may be partially attributable to differences in treatment recommendations for patients with bipolar versus unipolar depression. Although antidepressant medications are indicated for patients with bipolar depression, this may be stopped and replaced with a mood stabiliser or antipsychotic in patients that develop mania or hypomania.^42^ This, plus evidence that depressive episodes in bipolar disorder may be shorter than those in unipolar depression,^43^ could result in lower MSM scores. This association was not found in the PROMPT cohort in which ~27% of patients had bipolar disorder, meaning that these differences may only become relevant in patients with a more severe treatment-resistant illness. However, it could also relate to differences in the assessment of bipolar disorder between cohorts, as the PROMPT cohort was assessed using the MINI, which could result in different diagnoses in comparison with the full psychiatric assessments used for the ADU cohort. Psychotic symptoms have been associated with poorer outcomes in depression,^18^ and the results presented here suggest that within a cohort with established TRD, psychosis is associated with more severe resistance. However, the presence of current psychotic symptoms does contribute to the MSM severity subscale, and therefore may partially explain the association seen here.^11^

### Characteristics associated with the number of treatment failures

The number of previous episodes was positively associated with the number of antidepressant treatment failures in patients referred for psychological therapy, yet it was negatively associated in TRD out-patients and showed no association with the number of treatment failures in in-patients with the most severe history of treatment resistance. No other variables showed an association with the number of treatment failures in any of the multiple regression analyses. These findings suggest that recurrence does not have a consistent relationship with TRD severity, when considered purely as the number of treatment failures in the current episode. It may be that severity of previous episodes, which may differ between cohorts (and could not be assessed), contributes to the relationship between recurrence and resistance.

As discussed, all cohorts had similar proportions of participants with ≥3 previous episodes, but the PROMPT cohort had a lower number of current episode antidepressant treatment trials (mean 0.0, interquartile range (IQR) = 1.0). By contrast, the LQD study had an inclusion requirement of ≥2 trials in the current episode (mean 3.0, IQR = 2.0). Therefore, when treatment resistance is considered univariately as the number of treatment failures there are clear differences between patients above the much used ≥2 antidepressant treatment failures cut-off, and those below it. Interestingly, this finding supports the concept of patients with TRD being a distinct clinical group with different characterising features, when considered as a univariate construct. Further, the absence of an association between recurrence and number of antidepressant treatment trials in the ADU cohort suggests that the patients whose condition was the most severely resistant may also represent a distinct clinical group, and the severity of resistance does not increase with each subsequent episode of depression across the broader spectrum of TRD.

The only other association between antidepressant treatment trials and patient characteristics was in the univariate assessment of lifetime psychosis in the ADU cohort, which was negatively associated with antidepressant treatment trials, meaning patients in this cohort with lifetime psychosis had a less extensive history of suboptimal antidepressant responses, but this effect did not hold when other predictor variables were included in the model, suggesting an interaction between them. This univariate association is in the opposite direction to the association between lifetime psychosis and MSM score in the ADU cohort multiple regression (model 5), in which patients with lifetime psychosis had a higher degree of treatment resistance. This may in part be accounted for by the inclusion of psychotic symptoms in the MSM score, but may also suggest that there is an interaction between psychosis and other variables when predicting the number of antidepressant treatment failures (although multicollinearity was not deemed problematic) and therefore the link between a history of psychosis and different characterisations of TRD warrants further exploration. It is also worth noting that there were some differences in the assessment of lifetime psychosis. Thus, in the ADU cohort, the proportion with lifetime psychosis was relatively high (38.2%) and all psychosis occurred in the context of a mood episode as other cases of psychosis were excluded; in contrast, in the LQD cohort current psychosis was excluded and there were no identified individuals with lifetime psychosis. In the PROMPT cohort, the proportion with lifetime psychosis was relatively low (10.9%), and although the large majority of those participants (over 70%) scored positive for a history of psychotic symptoms in association with a mood disorder, the nature of the MINI assessment did not allow accurate retrospective diagnosis for these individuals.

### The measurement of TRD

Comparison of the characteristics associated with the multidimensional MSM score versus the one-dimensional number of treatment failures suggests that for moderate, established TRD (the LQD cohort), both are similarly associated with patient characteristics. However, associations differ between the MSM and antidepressant failure scores in the PROMPT and ADU cohorts. In PROMPT this is likely to relate to the low variance in antidepressant treatment failures and high incidence of patients who are pharmacologically treatment naive. However, in the ADU cohort reasons for the differences are less clear, and it is possible that the two measures of treatment resistance are capturing slightly different patient groups in this cohort. However, the greater number of characteristics significantly associated with MSM score indicates that it may have greater utility for identifying patients at risk of severe TRD. When considered with evidence that MSM score, but not the raw number of treatment trials, was predictive of subsequent treatment outcome in previous work^44^ it appears that identifying risk of TRD severity when characterised multidimensionally according to the MSM is more achievable and more clinically useful than unidimensional assessments of TRD.

### Limitations

First, the data-sets examined in the present work were not combined because of the extent of differences in their characteristics, treatment modalities received, study designs and relevant measures collected. Therefore, differences in the characteristics associated with severity of treatment resistance between cohorts may be confounded by some of these divergent factors, including the presence or absence of bipolarity and psychosis. Second, this work is retrospective, meaning the data is cross-sectional. Although highly dynamic characteristics (such as illness severity) were deliberately not included in the models, the results presented here need replication in a prospectively designed longitudinal study in which patients are followed up over the course of their illness in order for the associations with treatment resistance severity to be confirmed.

### Implications

This study finds that several patient characteristics are linearly associated with the severity of TRD when characterised as a multidimensional continuous construct via the MSM, and unidimensionally as a raw number of suboptimal antidepressant responses, although the majority of reported associations were not replicated across cohorts. Multidimensional characterisations of TRD may be more useful for the purpose of identifying TRD risk, but differences between cohorts suggest that characteristics may not be consistently related to severity of resistance across the TRD spectrum. Further exploration of the relationships between patient characteristics and MSM score using longitudinal data may provide greater insight into indicators of risk for the more severe forms of treatment resistance and should be one area of future investigation in order for this work to support the prioritisation of specialist treatments and the improvement of treatment outcomes for patients with depression.

## Data Availability

The data that support the findings of this study are available from the corresponding author (A.J.C.) upon reasonable request.

## Supplementary material

For supplementary material accompanying this paper visit http://dx.doi.org/10.1192/bjo.2021.1004. click here to view supplementary material
