# Supplementary material for: Characterising the severity of treatment resistance in unipolar and bipolar depression
Source: BJPsych Open. 2021 Oct 13;7(6):e193. doi: 10.1192/bjo.2021.1004 (PMC8517851; doi:10.1192/bjo.2021.1004)
Supplement: Supplementary file 1 [file S2056472421010048sup001.docx]

Characterising the severity of treatment resistant depression: Supplementary information


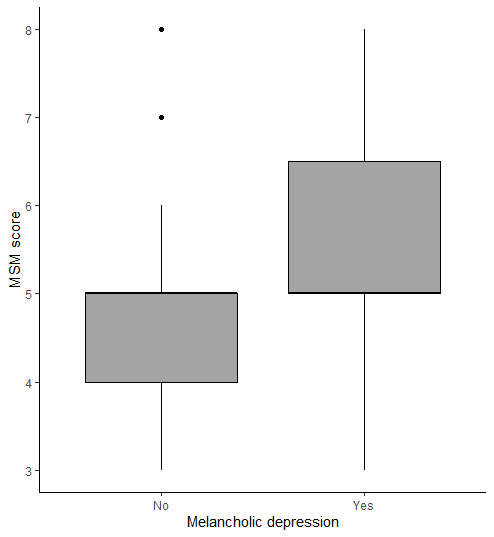
Supplementary Figures

Supplementary Figure 1. Association between melancholic depression and MSM score in the PROMPT study sample


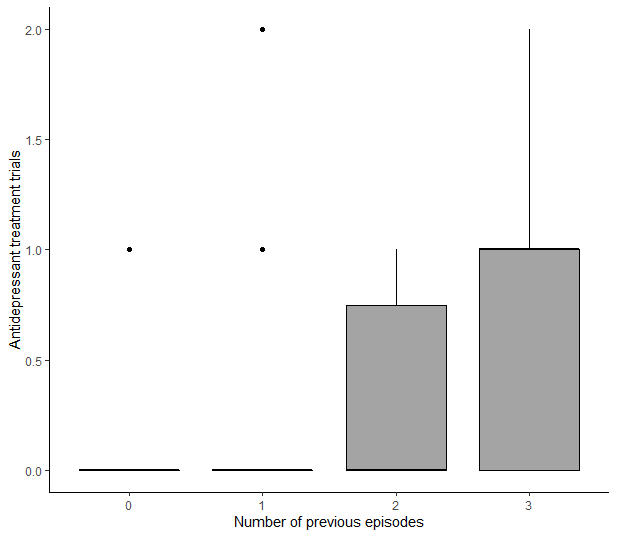


Supplementary figure 2. Association between number of depressive episodes and antidepressant treatment trials in the PROMPT sample


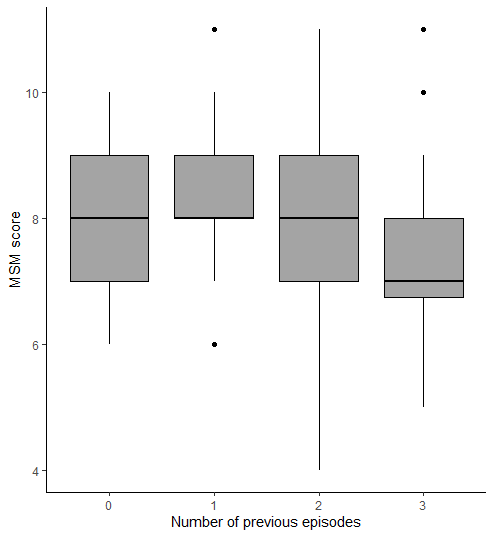


Figure 3. Boxplot of associations between number of previous episodes and MSM score in the LQD study sample


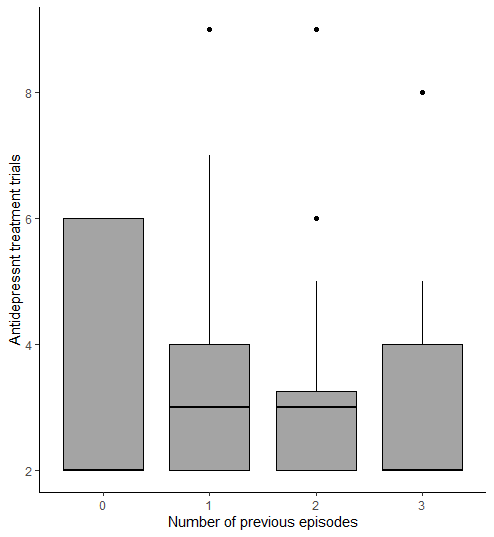


Supplementary Figure 4. Plot showing association between number of previous episodes and number of antidepressant treatment failures in the LQD study sample

Supplementary Tables

Supplementary Table 1. VIFs for PROMPT multiple regressions

|  | Dataset | | | | | | | | | |
| --- | --- | --- | --- | --- | --- | --- | --- | --- | --- | --- |
|  | MSM outcome | | | | | Antidepressants outcome | | | | |
|  | 1 | 2 | 3 | 4 | 5 | 1 | 2 | 3 | 4 | 5 |
| Number of previous episodes | 1.55 | 1.61 | 1.64 | 1.64 | 1.60 | 1.40 | 1.48 | 1.42 | 1.45 | 1.40 |
| Age of depression onset | 2.49 | 2.47 | 2.42 | 2.42 | 2.36 | 2.26 | 2.25 | 2.25 | 2.20 | 2.19 |
| Childhood trauma (CTQ) | 1.50 | 1.52 | 1.58 | 1.49 | 1.48 | 1.51 | 1.45 | 1.52 | 1.47 | 1.46 |
| Personality (SAPAS) | 1.84 | 1.79 | 1.80 | 1.77 | 1.83 | 1.63 | 1.63 | 1.67 | 1.72 | 1.64 |
| Sex (male) | 1.11 | 1.12 | 1.10 | 1.11 | 1.13 | 1.09 | 1.10 | 1.11 | 1.11 | 1.12 |
| Psychiatric comorbidities | 1.40 | 1.38 | 1.41 | 1.41 | 1.41 | 1.36 | 1.36 | 1.37 | 1.38 | 1.37 |
| Bipolar depression | 1.50 | 1.54 | 1.48 | 1.49 | 1.48 | 1.41 | 1.41 | 1.39 | 1.39 | 1.37 |
| Marital group | 1.69 | 1.61 | 1.61 | 1.66 | 1.70 | 1.50 | 1.53 | 1.56 | 1.48 | 1.44 |
| Physical illness (CIRS) | 1.40 | 1.38 | 1.36 | 1.35 | 1.41 | 1.24 | 1.27 | 1.29 | 1.29 | 1.27 |
| Duration of depressive illness | 1.99 | 2.03 | 2.04 | 2.00 | 2.05 | 1.99 | 2.01 | 2.01 | 2.02 | 1.97 |
| Lifetime psychosis | 1.26 | 1.31 | 1.30 | 1.30 | 1.26 | 1.22 | 1.22 | 1.23 | 1.22 | 1.21 |
| Melancholic depression | 1.26 | 1.27 | 1.22 | 1.26 | 1.22 | 1.19 | 1.19 | 1.18 | 1.18 | 1.18 |
| Education | 1.44 | 1.44 | 1.40 | 1.41 | 1.28 | 1.35 | 1.35 | 1.37 | 1.33 | 1.34 |

*VIF – Variance Inflation Factors, CTQ- childhood Trauma Questionnaire, SAPAS – Standardised Assessment of Personality – Abbreviated Scale, CIRS – Cumulative Illness Rating Scale*

Supplementary Table 2. VIFs for LQD multiple regressions

|  | Dataset | | | | | | | | | |
| --- | --- | --- | --- | --- | --- | --- | --- | --- | --- | --- |
|  | MSM outcome | | | | | Antidepressants outcome | | | | |
|  | 1 | 2 | 3 | 4 | 5 | 1 | 2 | 3 | 4 | 5 |
| Sex | 1.11 | 1.10 | 1.10 | 1.11 | 1.10 | 1.11 | 1.10 | 1.11 | 1.10 | 1.11 |
| Years of education | 1.23 | 1.22 | 1.23 | 1.23 | 1.22 | 1.21 | 1.21 | 1.22 | 1.22 | 1.21 |
| Age of onset | 1.58 | 1.59 | 1.59 | 1.58 | 1.58 | 1.58 | 1.59 | 1.58 | 1.59 | 1.60 |
| Duration of illness | 1.45 | 1.44 | 1.47 | 1.46 | 1.46 | 1.47 | 1.46 | 1.49 | 1.48 | 1.47 |
| Number of previous episodes | 1.12 | 1.10 | 1.12 | 1.13 | 1.12 | 1.13 | 1.11 | 1.13 | 1.12 | 1.12 |
| Psychiatric comorbidities (MINI) | 1.18 | 1.19 | 1.19 | 1.19 | 1.18 | 1.19 | 1.19 | 1.19 | 1.18 | 1.19 |
| Physical comorbidity | 1.04 | 1.03 | 1.04 | 1.04 | 1.04 | 1.04 | 1.04 | 1.04 | 1.05 | 1.05 |
| Martial group | 1.19 | 1.19 | 1.21 | 1.20 | 1.20 | 1.23 | 1.23 | 1.22 | 1.22 | 1.23 |
| Personality (SAPAS) | 1.18 | 1.19 | 1.18 | 1.20 | 1.18 | 1.19 | 1.19 | 1.19 | 1.18 | 1.20 |
| Atypical depression (IDS) | 1.09 | 1.10 | 1.10 | 1.09 | 1.09 | 1.10 | 1.11 | 1.10 | 1.11 | 1.11 |

*VIF – Variance Inflation Factors, MINI – MINI International Neuropsychiatric Interview, IDS – Inventory of Depressive Symptomatology, SAPAS – Standardised Assessment of Personality Abbreviated Version.*

Supplementary Table 3. VIFs for ADU multiple regressions

|  | Dataset | | | | | | | | | |
| --- | --- | --- | --- | --- | --- | --- | --- | --- | --- | --- |
|  | MSM outcome | | | | | Antidepressants outcome | | | | |
|  | 1 | 2 | 3 | 4 | 5 | 1 | 2 | 3 | 4 | 5 |
| Years of education | 1.11 | 1.11 | 1.11 | 1.13 | 1.10 | 1.09 | 1.11 | 1.13 | 1.10 | 1.11 |
| Age of onset | 2.15 | 2.18 | 2.17 | 2.13 | 2.18 | 2.18 | 2.20 | 2.22 | 2.19 | 2.20 |
| Lifetime psychosis | 1.06 | 1.07 | 1.07 | 1.08 | 1.07 | 1.06 | 1.08 | 1.09 | 1.08 | 1.07 |
| Family history | 1.15 | 1.12 | 1.12 | 1.12 | 1.14 | 1.13 | 1.11 | 1.11 | 1.12 | 1.13 |
| Childhood trauma | 1.09 | 1.08 | 1.08 | 1.09 | 1.10 | 1.09 | 1.10 | 1.09 | 1.08 | 1.08 |
| Bipolar/unipolar depression | 1.52 | 1.51 | 1.52 | 1.51 | 1.54 | 1.53 | 1.49 | 1.52 | 1.53 | 1.54 |
| Number of previous episodes | 1.90 | 1.88 | 1.91 | 1.91 | 1.92 | 1.90 | 1.88 | 1.89 | 1.88 | 1.88 |
| Psychiatric comorbidities | 1.17 | 1.14 | 1.14 | 1.13 | 1.15 | 1.15 | 1.13 | 1.16 | 1.14 | 1.15 |
| Duration of illness | 2.05 | 2.07 | 2.05 | 2.06 | 2.07 | 2.02 | 2.02 | 2.03 | 2.04 | 2.05 |
| Sex | 1.14 | 1.11 | 1.12 | 1.10 | 1.11 | 1.14 | 1.14 | 1.11 | 1.11 | 1.14 |
| Marital group | 1.43 | 1.45 | 1.43 | 1.45 | 1.24 | 1.41 | 1.39 | 1.40 | 1.37 | 1.41 |
| Physical comorbidity | 1.12 | 1.12 | 1.09 | 1.10 | 1.12 | 1.13 | 1.13 | 1.12 | 1.10 | 1.16 |

*VIF – Variance Inflation Factors*

Supplementary Table 4. Participant characteristics – Antidepressants outcome

|  |  | PROMPT | | | LQD^a^ | | | ADU | | |
| --- | --- | --- | --- | --- | --- | --- | --- | --- | --- | --- |
|  | Unit | N | Median(IQR) | % | N | Median(IQR) | % | N | Median(IQR) | % |
| Age | Years |  | 36.0(21.0) |  |  | 42.9(22.2) |  |  | 50.0(18.5) | - |
| Sex | Female Male | 149 |  | 67.8  32.2 | 199 | - | 45.2  54.8 | 180 |  | 74.4  25.6 |
| Ethnicity | White Black Asian Other | 116 |  | 71.6  3.5  11.2  13.8 | 198 | - | 90.5  2.0  4.0  3.5 | 176 |  | 97.2  1.1  1.7  0.0 |
| Marital group ^b:^ | Single Separated Steady | 142 |  | 51.4  8.5  40.1 | 198 | - | 46.5  8.6  45.0 |  |  | 21.1  61.7  17.2 |
| Education | Categories ^c^ (PROMPT) or years (LQD, ADU | 142 |  | 1: 11.3  2: 12.7  3: 21.8  4: 54.2 | 199 | 15.0(3.5) | - | 175 | 13.0(5.0) | - |
| Age at onset | Years | 149 | 16.0(12.0) | - | 199 | 17.0(15.0) | - | 179 | 27.0(23.0) | - |
| Duration of illness | Years | 149 | 16.0(16.0) | - | 199 | 18.8(17.5) | - | 179 | 15.0(20.5) | - |
| N. previous episodes | 0 1 2 ≥3 | 149 |  | 28.9  14.6  7.4  49.0 | 189 | - | 2.7  34.9  21.2  41.3 | 176 | - | 22.1  18.2  12.5  47.2 |
| Diagnosis | Unipolar Bipolar | 149 |  | 72.5  27.5 | 199 | - | 100.0  0.0 | 180 | - | 71.7  28.3 |
| Melancholic subtype | No Yes | 142 |  | 50.0  50.0 | - | - | - | - | - | - |
| Lifetime psychosis | No Yes | 148 |  | 89.9  10.1 | - | - | - | 179 | - | 62.0  38.0 |
| Atypical subtype | No Yes | - |  | - | 199 | - | 93.0  7.0 | - | - | - |
| N. Psychiatric comorbidities | N | 148 | 1.0(1.0) | - | 198 | 2.0(2.0) | - | 173 | 0.0(1.0) | - |
| Family history ^d^ | No Possible Definite |  |  |  |  |  |  | 170 |  | 40.6  14.1  45.3 |
| Physical illness | Yes  No | 148 | 15.0(4.0) ^e^ | - | 196 | - | 82.7  17.4 | 167 |  | 72.5  27.5 |
| Childhood trauma | Yes  No | 139 | 38.0(23.0) ^f^ | - | - | - | - | 173 |  | 60.1  39.9 |
| Personality (SAPAS) | Score | 142 | 3.0(3.0) | - |  | 4.0(2.0) |  |  |  |  |
| Antidepressant trials | Score | 149 | 0.0(1.0) | - |  | 3.0(2.0) | - | 180 | 6.0(4.0) |  |

*^a^ Participants recruited from sites in London (37%), North East (26%), Oxford (26%), Brighton (7%), Bristol (5%).* ^b^ *Marital group categorisation: Steady = long-term relationship, cohabiting, married; Separated = divorced, marriage separated, widowed; Single = otherwise. ^c^ Education categories, 1 = no qualifications 2=secondary 3=college 4= ≥Degree. ^d^ First degree relative with affective disorder. ^e^ Cumulative Illness Rating Scale score. ^f^ Childhood Trauma Questionnaire score. AD – antidepressant, MSM - Maudsley Staging Method, N – number, IQR – interquartile range, % - percentage, GCSE – General Certificate of Secondary Education, A level – Advanced level, CIRS – Cumulative Illness Rating Scale, SAPAS – Standardised Assessment of Personality - Abbreviated Scale.*

Supplementary Table 5. Univariate models for PROMPT, MSM outcome)

|  | β (S.E) | t | p | R2 |
| --- | --- | --- | --- | --- |
| Intercept  Sex (male) | 5.05(0.14)  0.18(0.25) | 36.61  0.73 | <0.001  0.468 | <0.01 |
| Intercept  Marital group ^a^ (separated)  Marital group ^a^ (steady) | 5.21(0.16)  -0.03(0.48)  -0.24(0.24) | 32.30  -0.06  -0.98 | <0.001  0.954  0.331 | 0.01 |
| Intercept  Education | 6.23(0.35)  -0.35(0.11) | 17.80  -3.38 | <0.001  <0.001** | 0.08 |
| Intercept  Age of depression onset | 5.05(0.23)  0.003(0.01) | 22.45  0.29 | <0.001  0.774 | <0.01 |
| Intercept  Duration of depressive illness | 4.90(0.19)  0.01(0.01) | 25.43  1.36 | <0.001  0.178 | 0.01 |
| Intercept  Number of previous episodes | 5.65(0.26)  -0.20(0.09) | 21.41  -2.27 | <0.001  0.025* | <0.01 |
| Intercept  Melancholic depression | 4.72(0.15)  0.81(0.22) | 30.74  3.65 | <0.001  <0.001** | 0.09 |
| Intercept  Psychiatric comorbidities | 5.00(0.15)  0.14(0.13) | 33.44  1.10 | <0.001  0.274 | 0.01 |
| Intercept  Physical illness (CIRS) | 2.98(0.62)  0.13(0.04) | 4.85  3.50 | <0.001  <0.001** | 0.08 |
| Intercept  Childhood trauma (CTQ) | 4.50(0.36)  0.01(0.01) | 12.50  1.78 | <0.001  0.079 | 0.03 |
| Intercept  Personality (SAPAS) | 4.51(0.26)  0.17(0.07) | 17.30  2.57 | <0.001  0.012* | 0.05 |
| Intercept  Bipolar depression | 5.05(0.13)  0.21(0.26) | 37.56  0.83 | <0.001  0.408 | 0.01 |
| Intercept  Lifetime psychosis | 5.03(0.12)  0.65(0.36) | 41.71  1.84 | <0.001  0.067 | 0.02 |

^a^ *Marital group categorisation: Steady = long-term relationship, cohabiting, married; Separated = divorced, marriage separated, widowed; Single = otherwise. **P<0.01, β – beta coefficient, S.E – standard error, t – test statistic, p – significance, CTQ- childhood Trauma Questionnaire, SAPAS – Standardised Assessment of Personality – Abbreviated Scale, CIRS – Cumulative Illness Rating Scale.*

Supplementary Table 6. Univariate models for PROMPT, antidepressants outcome

|  | β (S.E) | t | p | R2 |
| --- | --- | --- | --- | --- |
| Intercept  Sex (male) | 0.37(0.05)  0.11(0.09) | 7.11  1.24 | <0.001  0.216 | 0.01 |
| Intercept  Marital group ^a^ (separated)  Marital group ^a^ (steady) | 0.45(0.06)  0.18(0.15)  -0.16(0.09) | 7.56  1.22  -1.68 | <0.001  0.225  0.096 | 0.04 |
| Intercept  Education | 0.47(0.14)  -0.02(0.04) | 3.36  -0.52 | 0.001  0.604 | <0.01 |
| Intercept  Age of depression onset | 0.44(0.08)  <-0.01(0<0.01) | 5.25  -0.48 | <0.001  0.629 | <0.01 |
| Intercept  Duration of depressive illness | 0.30(0.07)  0.01(<0.01) | 4.18  1.75 | <0.001  0.082 | 0.02 |
| Intercept  Number of previous episodes | 0.19(0.10)  0.08(0.03) | 1.93  2.45 | 0.055  0.016* | <0.04 |
| Intercept  Melancholic depression | 0.34(0.06)  0.12(0.09) | 5.72  1.44 | <0.001  0.153 | 0.01 |
| Intercept  Psychiatric comorbidities | 0.40(0.06)  <-0.01(0.05) | 7.26  -0.05 | <0.001  0.958 | <0.01 |
| Intercept  Physical illness (CIRS) | 0.15(0.23)  0.02(0.01) | 0.63  1.15 | 0.527  0.253 | 0.01 |
| Intercept  Childhood trauma (CTQ) | 0.33(0.13)  <0.01(<0.01) | 2.59  0.63 | 0.011  0.529 | <0.01 |
| Intercept  Personality (SAPAS) | 0.35(0.10)  0.02(0.03) | 3.55  0.66 | <0.001  0.509 | <0.01 |
| Intercept  Bipolar depression | 0.40(0.05)  0.02(0.10) | 7.95  0.17 | <0.001  0.863 | <0.01 |
| Intercept  Lifetime psychosis | 0.42(0.04)  -0.15(0.14) | 9.33  -1.07 | <0.001  0.286 | 0.01 |

^a^ *Marital group categorisation: Steady = long-term relationship, cohabiting, married; Separated = divorced, marriage separated, widowed; Single = otherwise. *P<0.05, , β – beta coefficient, S.E – standard error, t – test statistic, p – significance, CTQ- childhood Trauma Questionnaire, SAPAS – Standardised Assessment of Personality – Abbreviated Scale, CIRS – Cumulative Illness Rating Scale.*

Supplementary Table 7. Univariate models for LQD, MSM outcome

|  | β (S.E) | t | p | R2 |
| --- | --- | --- | --- | --- |
| Intercept  Sex | 7.79(0.17)  0.04(0.20) | 46.21  0.19 | <0.001  0.847 | <0.01 |
| Intercept  Divorced/separated  Married/civil partnership | 7.83(0.17)  0.72(0.36)  -0.17(0.20) | 46.86  1.98  -0.81 | <0.001  0.048*  0.417 | 0.03 |
| Intercept  Years of education | 7.80(0.53)  <0.01(0.04) | 14.70  0.03 | <0.001  0.979 | <0.01 |
| Intercept  Age of onset | 8.22(0.21)  -0.02(0.01) | 38.42  -2.44 | <0.001  0.015* | 0.03 |
| Intercept  Duration of illness | 7.59(0.20)  0.01(0.01) | 37.92  1.34 | <0.001  0.179 | 0.01 |
| Intercept  Number of previous episodes | 9.19(0.34)  -0.46(0.10) | 27.15  -4.43 | <0.001  <0.001** | 0.09 |
| Intercept  Atypical depression | 7.82(0.13)  -0.19(0.37) | 59.48  -0.51 | <0.001  0.610 | <0.01 |
| Intercept  Psychiatric comorbidity | 7.58(0.17)  0.11(0.06) | 45.95  1.86 | <0.001  0.063 | 0.02 |
| Intercept  Physical comorbidity | 7.58(0.25)  0.27(0.26) | 30.11  1.02 | <0.001  0.306 | 0.01 |
| Intercept  Personality | 7.88(0.32)  -0.02(0.07) | 24.64  0.07 | <0.001  0.815 | <0.01 |

**p<0.05, **p<0.01, β – beta coefficient, S.E – standard error, t – test statistic, p – significance*

Supplementary Table 8. Univariate models for LQD, antidepressant outcome

|  | β (S.E) | t | p | R2 |
| --- | --- | --- | --- | --- |
| Intercept  Sex | 3.11(0.21)  0.20(0.19) | 15.12  1.01 | <0.001  0.311 | <0.01 |
| Intercept  Divorced/separated  Married/civil partnership | 3.16(0.21)  0.55(0.36)  0.05(0.20) | 15.14  1.54  0.23 | <0.001  0.124 | 0.01 |
| Intercept  Years of education | 2.81(0.53)  0.03(0.04) | 5.27  0.81 | <0.001  0.421 | <0.01 |
| Intercept  Age of onset | 3.37(0.24)  -0.01(0.01) | 13.86  -0.94 | <0.001  0.347 | <0.01 |
| Intercept  Duration of illness | 3.24(0.24)  <-0.01(0.01) | 13.52  -0.12 | <0.001  0.907 | <0.01 |
| Intercept  Number of previous episodes | 4.32(0.35)  -0.37(0.10) | 12.24  -3.62 | <0.001  <0.001* | 0.06 |
| Intercept  Atypical depression | 3.21(0.18)  0.21(0.38) | 17.67  0.56 | <0.001  0.574 | <0.01 |
| Intercept  Psychiatric comorbidity | 3.29(0.21)  -0.04(0.06) | 15.49  -0.63 | <0.001  0.528 | <0.01 |
| Intercept  Physical comorbidity | 3.11(0.28)  0.13(0.26) | 11.17  0.50 | <0.001  0.618 | <0.01 |
| Intercept  Personality | 3.69(0.33)  -0.11(0.07) | 11.09  -1.67 | <0.001  0.094 | 0.01 |

**p<0.05, β – beta coefficient, S.E – standard error, t – test statistic, p – significance*

Supplementary Table 9. Univariate models for linear model 5 (ADU, MSM outcome)

|  | β (S.E) | t | p | R2 |
| --- | --- | --- | --- | --- |
| Intercept  Sex (Female) | 10.93(0.31)  -0.16(0.36) | 35.39  -0.45 | <0.001  0.652 | <0.01 |
| Intercept  Divorced/separated/widowed  Married/cohabiting | 10.12(0.35)  0.75(0.40)  1.31(0.51) | 29.27  1.90  2.55 | <0.001  0.059  0.012* | 0.04 |
| Intercept  Years of education | 12.18(0.62)  -0.10(0.04) | 19.63  -2.27 | <0.001  0.025* | 0.03 |
| Intercept  Age of onset | 10.21(0.36)  0.02(0.01) | 28.46  1.87 | <0.001  0.064 | 0.02 |
| Intercept  Duration of illness | 10.64(0.27)  0.01(0.11) | 39.43  0.80 | <0.001  0.426 | <0.01 |
| Intercept  Number of previous episodes | 10.96(0.39)  -0.05(0.13) | 27.74  -0.40 | <0.001  0.692 | <0.01 |
| Intercept  Number of psychiatric comorbidities | 10.73(0.18)  0.19(0.22) | 58.05  0.84 | <0.001  0.401 | <0.01 |
| Intercept  Presence of physical comorbidity | 10.62(0.30)  0.27(0.36) | 35.28  0.76 | <0.001  0.451 | <0.01 |
| Intercept  Presence of childhood trauma | 11.08(0.25)  -0.45(0.32) | 44.75  -1.39 | <0.001  0.168 | 0.01 |
| Intercept  Bipolar depression | 11.07(0.18)  -0.93(0.34) | 60.96  -2.71 | <0.001  0.007** | 0.04 |
| Intercept  Presence of lifetime psychosis | 10.61(0.20)  0.53(0.32) | 53.46  1.65 | <0.001  0.101 | 0.02 |

**p<0.05, **p<0.01, β – beta coefficient, S.E – standard error, t – test statistic, p – significance*

Supplementary Table 10. Univariate models for linear model 6 (ADU, antidepressants outcome)

|  | β (S.E) | t | p | R2 |
| --- | --- | --- | --- | --- |
| Intercept  Sex (Female) | 5.89(0.50)  0.27(0.58) | 11.81  0.47 | <0.001  0.638 | <0.01 |
| Intercept  Divorced/separated/widowed  Married/cohabiting | 5.21(0.55)  1.01(0.63)  1.50(0.81) | 9.56  1.61  1.84 | <0.001  0.120  0.067 | 0.02 |
| Intercept  Years of education | 7.71(1.00)  -0.12(0.07) | 7.74  -1.68 | <0.001  0.095 | 0.02 |
| Intercept  Age of onset | 5.80(0.58)  0.01(0.02) | 10.03  0.56 | <0.001  0.574 | <0.01 |
| Intercept  Duration of illness | 5.69(0.43)  0.02(0.02) | 13.11  1.14 | <0.001  0.256 | <0.01 |
| Intercept  Number of previous episodes | 5.71(0.64)  0.13(0.21) | 8.93  0.65 | <0.001  0.515 | <0.01 |
| Intercept  Number of psychiatric comorbidities | 6.05(0.30)  0.09(0.35) | 20.30  0.25 | <0.001  0.805 | <0.01 |
| Intercept  Presence of physical comorbidity | 5.54(0.48)  0.78(0.56) | 11.60  1.37 | <0.001  0.171 | 0.01 |
| Intercept  Presence of childhood trauma | 6.63(0.39)  -0.90(0.51) | 16.81  -1.76 | <0.001  0.080 | 0.02 |
| Intercept  Bipolar depression | 6.35(0.30)  -0.90(0.56) | 21.45  -1.61 | <0.001  0.108 | 0.01 |
| Intercept  Presence of lifetime psychosis | 6.47(0.32)  -1.00(0.51) | 20.42  -1.95 | <0.001  0.053 | 0.02 |

**p<0.05, β – beta coefficient, S.E – standard error, t – test statistic, p – significance*
